# Clinical Outcomes After Proton Therapy Reirradiation for Recurrent Malignant Glioma: Analysis From the Prospective Proton Collaborative Group Registry

**DOI:** 10.1016/j.adro.2025.101834

**Published:** 2025-06-13

**Authors:** Omer Gal, Stephen Mihalcik, Lia M. Halasz, John H. Chang, C. Jake Wang, J. Isabelle Choi, Charles B. Simone, Carlos E. Vargas, Henry K. Tsai, Rupesh Kotecha, Robert H. Press

**Affiliations:** a Department of Radiation Oncology, Miami Cancer Institute, Miami, Florida; b Department of Radiation Oncology, Northwestern Medicine Cancer Center Warrenville and Northwestern Medicine Proton Center, Warrenville, Chicago, Illinois; c Department of Radiation Oncology, University of Washington, Seattle, Washington; d Department of Radiation Oncology, The Oklahoma Proton Center, Oklahoma City, Oklahoma; e Department of Radiation Oncology, Willis-Knighton Proton Therapy Center, Shreveport, Louisiana; f Department of Radiation Oncology, New York Proton Center, New York, New York; g Department of Radiation Oncology, Mayo Clinic Arizona, Phoenix, Arizona; h Department of Radiation Oncology, ProCure Proton Therapy Center, Somerset, New Jersey

## Abstract

**Purpose:**

Optimal treatment for recurrent glioma after prior radiation therapy (RT) is not well established. Proton therapy (PT) is increasingly used for reirradiation (ReRT); however, treatment outcomes, toxicities, and prognostic factors for PT-ReRT remain poorly defined.

**Methods and Materials:**

The prospective, multi-institutional Proton Collaborative Group registry was queried for patients with malignant glioma who underwent PT-ReRT between July 2011 and December 2023; only patients with at least one follow-up encounter were included. Overall survival (OS) and progression-free survival were assessed using the Kaplan-Meier method, and Cox proportional hazards regression was used for uni- and multivariable analyses (univariable analysis and multivariable analysis).

**Results:**

The study cohort included 143 patients, the median follow-up was 11.2 months, and the median time interval (TI) from prior RT (median 58.5 Gy, IQR, 54-60 Gy) to PT-ReRT (median 44.6 Gy, IQR, 39.4-55.9 Gy) was 42.4 months. Median progression-free survival and OS were 8.1 and 11.2 months, respectively. On univariable analysis, improved OS was associated with oligodendroglioma and astrocytoma histology compared to glioblastoma, TI >60 months, Eastern Cooperative Oncology Group performance status 0, and ReRT dose ≥50 Gy. On multivariable analysis, improved OS remained associated only with oligodendroglioma and TI >60 months. Acute and late grade 3 toxicity occurred in 7% and 4%, respectively. Acute grade 3 toxicity was associated with poor performance status. Incidence of radiographic radiation necrosis was 19%.

**Conclusions:**

In the largest series of glioma PT-ReRT reported to date, retreatment was well tolerated with variable outcomes based on clinical prognostic factors. Toxicity rates were similar compared to photon-based literature despite a high median ReRT prescription dose.

## Introduction

Malignant gliomas are a distinct entity representing 25% of all primary brain tumors. World Health Organization (WHO) grade 4 glioblastomas comprise 57% of cases, while WHO grade 2 to 3 astrocytomas and oligodendrogliomas account for another 21%.^1^ Despite standard upfront multimodal therapies, malignant glioma inevitably recurs, most commonly in-field and/or marginal to the original resection bed and radiation therapy (RT) treatment fields.^2^^,^^3^ In the recurrent setting, outcomes and optimal salvage therapies are not well established. Treatment options include maximally safe re-resection, systemic therapies, tumor treating fields, and reirradiation (ReRT).^4^ In certain patients, a second course of RT can be considered with the goal of providing durable disease control and preventing or delaying the associated morbidity of local tumor progression.

Reirradiation of recurrent glioma can be challenging due to the previous receipt of high-dose RT in close proximity to critical central nervous system (CNS) organs-at-risk (OARs), such as the brainstem and optic structures. Excess cumulative doses to CNS OARs and normal nontarget brain tissue may increase the risk of significant neurologic sequela and are detrimental to patient quality of life.^5^, ^6^, ^7^, ^8^, ^9^ As such, administration of definitive ReRT doses with photon therapy is not typically feasible due to risks of high-grade toxicities.

Proton therapy (PT) is an increasingly used treatment modality for ReRT.^10^ The physical properties of PT compared to photon therapy offer greater capability to treat recurrent disease with meaningful radiation doses while minimizing dose to normal tissues within the brain by eliminating exit-dose beyond the target volume. Therefore, PT has potential in the recurrent setting; however, prospective clinical data remain limited.^11^ In this context, the goal of this study was to analyze patients with recurrent glioma enrolled in the prospective, multi-institutional Proton Collaborative Group (PCG) registry for treatment patterns, patient outcomes, and treatment-related toxicities. Additionally, this study aimed to identify favorable prognostic factors, thus enabling the selection of patients with a longer-term prognosis who will more likely benefit from the reduced OARs exposure of PT-ReRT.

## Materials and Methods

The PCG is a research consortium of proton centers in the United States. REG001-09 (NCT01255748) is a prospective registry study for which each of 13 participating PCG institutions obtained individual institutional review board approval. The study opened for accrual in 2010 and is currently enrolling patients. The registry was queried for all consecutive patients with recurrent glioma treated with PT-ReRT from July 2011 and December 2023 and who had at least one follow-up visit following the completion of treatment. Patient and tumor characteristics, prior and current radiation details, clinical outcomes, and toxicities were extracted and reviewed retrospectively.

Patients were recommended for PT-ReRT at the discretion of each individual institutional multi-disciplinary tumor board. Overall survival (OS) and progression-free survival (PFS) were defined as time from initiation of PT-ReRT to the event of interest. These results were recorded individually by each institution and reviewed centrally by PCG staff. Toxicities were scored according to National Cancer Institute Common Terminology Criteria for Adverse Events version 4.0. To account for differences in dose fractionation, radiation dose was summed via equivalent dose in 2 Gy fractions (EQD2) using a representative *α*/*β* ratio of 10 and 2.5 for malignant tumor response and normal tissue toxicity, respectively.

### Statistical analysis

Descriptive statistics were used to report patient, tumor, and treatment characteristics. Outcomes were assessed using the Kaplan-Meier method and univariable Cox proportional hazards regression analysis, denoted as hazard ratios (HRs) with 95% CIs. Demographic and treatment details included in the univariable analysis (UVA) were age, self-reported sex, race, tumor location, histology, grade, isocitrate dehydrogenase (IDH) mutation status, 1p19q co-deletion, O6-methylguanine-DNA methyltransferase (MGMT) methylation status, Eastern Cooperative Oncology Group (ECOG) performance status, tumor size, surgical resection, extent of resection, systemic therapy use, RT dose and fractionation, and time interval from prior RT to PT-ReRT. Statistically significant variables were then considered in a multivariable analysis (MVA). Univariable binomial regression analysis was performed to identify clinical correlates of toxicity. The statistical program used was SPSS version 29.0. All statistical analyses were performed using a *P* value for statistical significance set at <.05.

## Results

Patient, tumor, and treatment characteristics are detailed in Table 1. The cohort included 143 patients with recurrent glioma treated with PT across 13 treatment centers. Of those, 55% were male, 73% were White, and the median age was 49 years (range, 19-85) for all patients, and 54 years for patients with glioblastoma (range, 24-85). Most patients had an ECOG performance status of 0 (*n* = 64) or 1 (*n* = 50). The original primary tumor histology was glioblastoma (*n* = 74), astrocytoma (*n* = 35, 54% grade 3), and oligodendroglioma (*n* = 34, 62% grade 3). Molecular data were available for only a minority of the patients; IDH1 mutation and MGMT methylation were present in 22% (*n* = 10 of 45. Histology was glioblastoma, astrocytoma, and oligodendroglioma in 6, 3, and 1 patients) and 53% of patients (*n* = 27 of 51. Histology was glioblastoma, astrocytoma, and oligodendroglioma in 25, 1, and 1 patients), respectively.

**Table 1:** Patient and treatment characteristics

Parameter	Level	*N* (%) = 143
Age (y)	Median (range)	49 (19-85)
Sex	Male	78 (55%)
	Female	65 (45%)
ECOG PS	0	64 (45%)
	1	50 (35%)
	2	21 (15%)
	3	8 (5%)
Histology at diagnosis	Glioblastoma	74 (52%)
	Astrocytoma	35 (24%)
	Oligodendroglioma	34 (24%)
Tumor grade	4	74 (52%)
	3	40 (28%)
	2	29 (20%)
Prior surgical resection	Yes	128 (90%)
	No	15 (10%)
Prior chemotherapy	Yes	125 (87%)
	No	18 (13%)
Surgical resection within 3 months of PT	Yes	40 (33%)
	No	83 (67%)
Prior RT dose (Gy)	Median (range)	58.5 (36.2-120.9)
Time interval from prior RT to PT (months)	Median (range)	42.4 (3.9-457.9)
PT reirradiation dose (Gy)	Median (range)	44.6 (24.8-60.1)
Fraction size (Gy)	Median (range)	2.2 (1.1-4.0)
PT modality	PBS	89 (62%)
	PS/US	54 (38%)
Cumulative dose (Gy)	Median (range)	101.6 (80.8-171.8)

*Abbreviations:* ECOG PS = Eastern Cooperative Oncology Group performance status; PBS = pencil beam scanning; PS/US = Passive Scatter/Uniform Scanning; PT = proton therapy; RT = radiation therapy.

**Figure 1:**
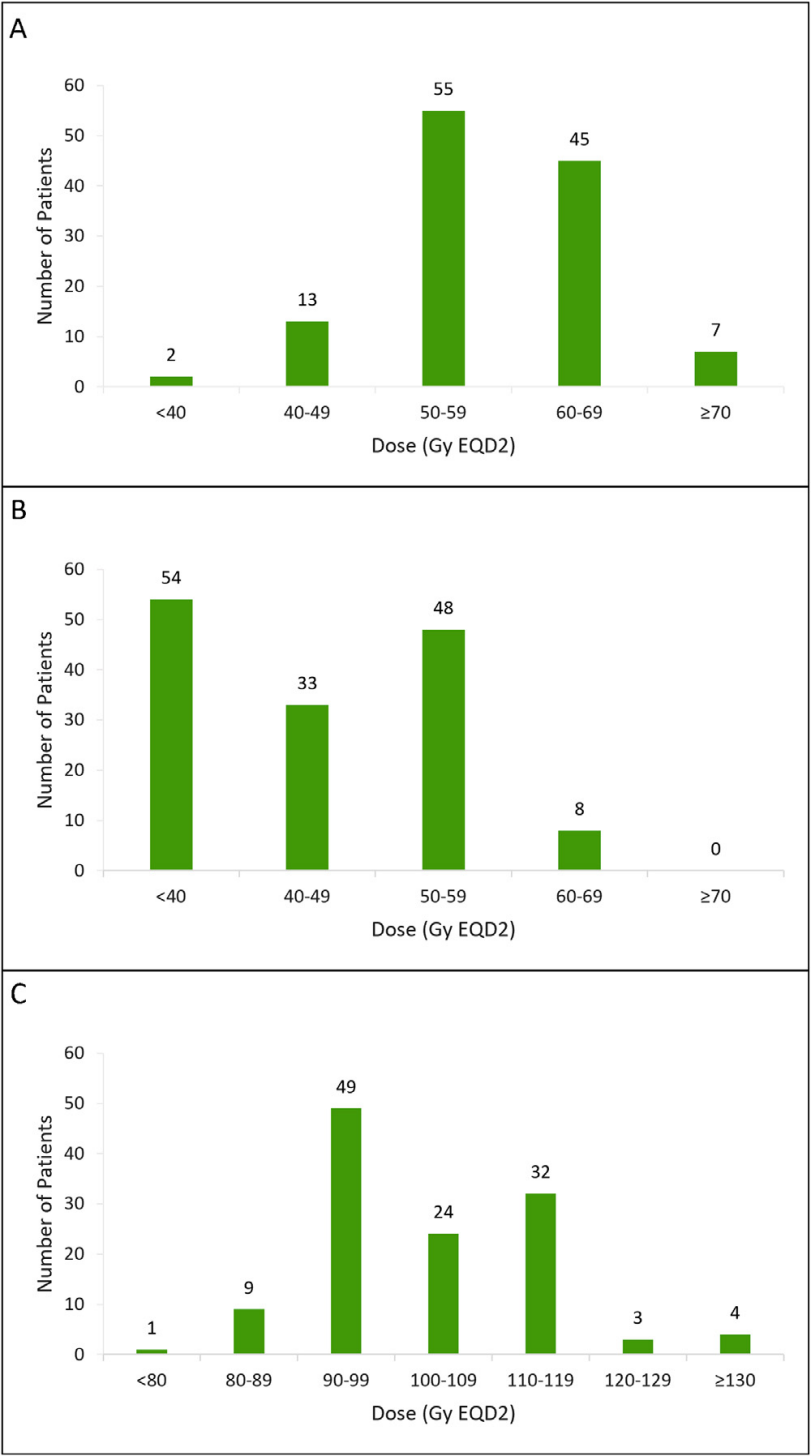
Cumulative prior radiation therapy dose (A), current reirradiation dose (B), and total cumulative dose (C). *Abbreviation:* EQD2 = equivalent dose in 2-Gy fractions.

Overall, 90% and 87% of patients had surgery and chemotherapy at initial diagnosis, respectively. Most patients received temozolomide (*n* = 103 of 125) or procarbazine, lomustine, and vincristine (PCV, *n* = 14). The median cumulative dose of the prior RT was 58.5 Gy (range, 36.2-120.9, IQR, 54-60 Gy). Most patients (89%) received 40 to 60 Gy given in one prior RT course, as illustrated in Fig. 1A; only 4 patients had more than one previous treatment course, and only 2 received less than 40 Gy. Most patients were originally treated using conventional fractionation (1.8-2.0 Gy/fraction); however, 9% received altered dose fractionation regimens (most commonly hypofractionated RT).

The median time from initial diagnosis to PT-ReRT was 62.5 months (range, 3.2–386.7 months). Respectively, 33% and 44% of patients had surgery 3 months and 6 months before PT-ReRT. The median time from prior RT to initiation of PT-ReRT for the entire cohort and specifically for patients with glioblastoma was 42.4 months (range, 3.9-457.9) and 15.7 months (range, 3.9-141.2), respectively. Pencil beam scanning PT (*n* = 89, 62%) was more commonly used than uniform scanning PT (*n* = 54, 38%). The median dose of PT-ReRT was 44.6 Gy (range, 24.8-60.1, IQR, 39.4-55.9 Gy, Fig. 1B) with a median dose/fraction size of 2.2 Gy fraction (range, 1.1-4.0). The median cumulative dose of all RT courses was 101.6 Gy (range, 80.8-171.8, Fig. 1C). Sixty-six percent of patients (94/143) had concurrent systemic therapy with PT-ReRT; the most common agents included temozolomide (*n* = 48), bevacizumab (*n* = 17), or temozolomide/bevacizumab combination (*n* = 20).

### Progression-free survival and overall survival

**Figure 2:**
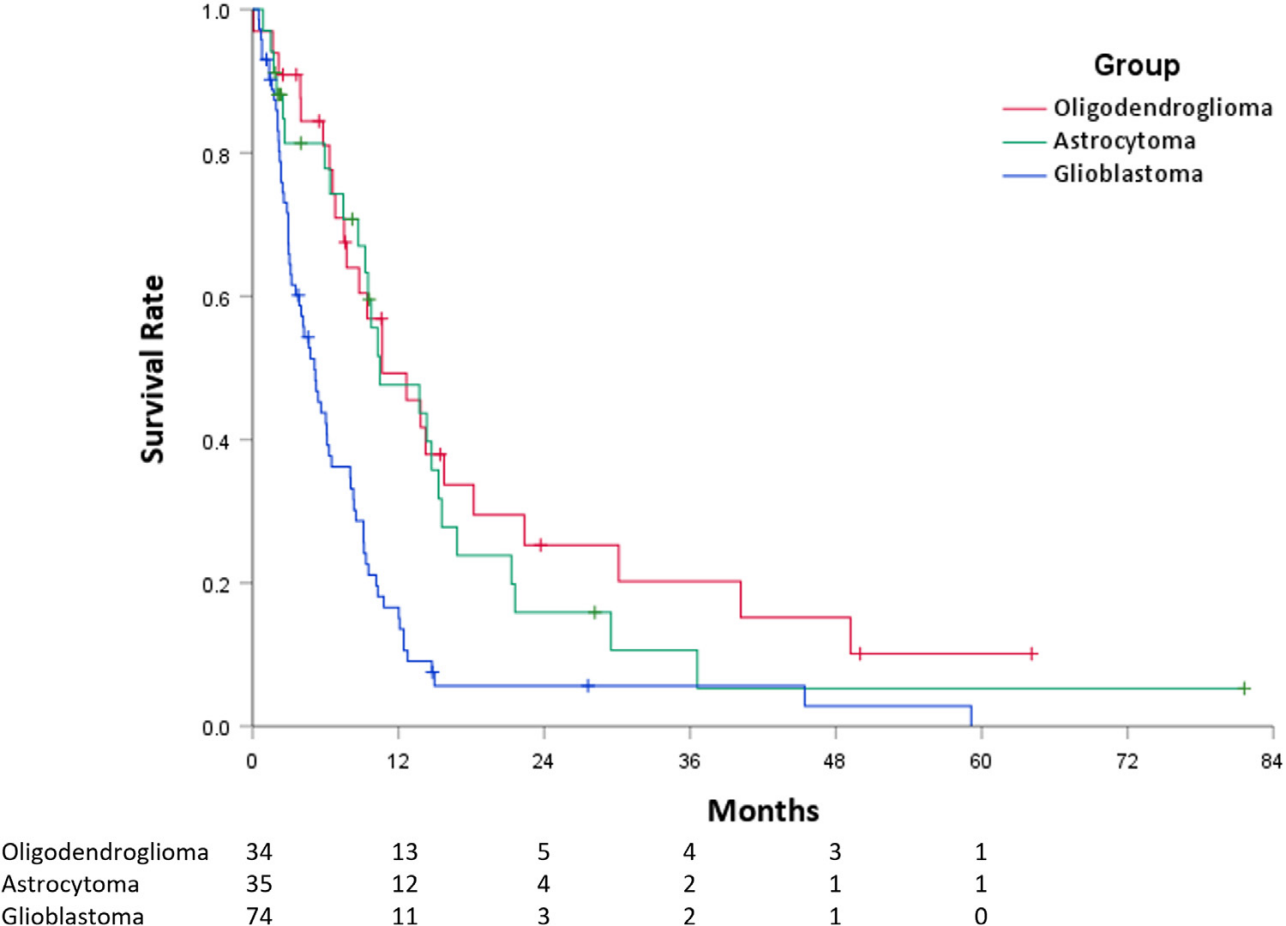
Kaplan-Meier curves demonstrating progression-free survival by original histology (red = oligodendroglioma, green = astrocytoma, blue = glioblastoma).

The median follow-up time for surviving patients after PT-ReRT was 11.2 months. The median PFS was 8.1 months (95% CI, 6.1-10.0). Median PFS was longer for oligodendroglioma (10.7 months, 95% CI, 5.4-16.0) and astrocytoma (10.5 months, 95% CI, 4.2-16.7) histologies than for glioblastoma (5.1 months, 95% CI, 3.7-6.5, *P* < .001) (Fig. 2). On UVA, improved PFS was associated with oligodendroglioma (HR 0.38, 95% CI, 0.24-0.62, *P* < .001) and astrocytoma (HR 0.457, 95% CI, 0.29-0.73, *P* = .001) histologies compared to glioblastoma, time interval since prior RT >60 months (HR 0.41, 95% CI, 0.26-0.66, *P* < .001), surgery within 3 months before PT-ReRT (HR 0.63, 95% CI, 0.41-0.97, *P* = .035), and ReRT dose ≥50 Gy (HR 0.66, 95% CI, 0.45-0.95, *P* = .027). On MVA, improved PFS remained significant for oligodendroglioma (HR 0.32, 95% CI, 0.17-0.60, *P* < .001) and astrocytoma (HR 0.34, 95% CI, 0.17-0.71, *P* = .004) histologies, and time since prior RT >60 months (HR 0.48, 95% CI, 0.29-0.80, *P* = .005) (Table 2).

**Table 2:** Univariable and multivariable analysis for progression-free survival and overall survival

		Univariable analysis		Multivariable analysis	
Variable	Level	HR (95% CI)	*P* value	HR (95% CI)	*P* value
PFS					
Histology	Oligodendroglioma	0.38 (0.24-0.62)	<.001	0.32 (0.17-0.60)	<.001
	Astrocytoma	0.57 (0.29-0.73)	<.001	0.34 (0.17-0.71)	.004
	Glioblastoma	REF		REF	
Time to recurrence	>60 mo	0.41 (0.26-0.66)	<.001	0.48 (0.29-0.80)	.005
	<60 mo	REF		REF	
Surgery within 3 mo	Yes	0.63 (0.41-0.97)	.035		NS
	No	REF			
PT-ReRT dose	≥50 Gy	0.66 (0.45–0.95)	.027		NS
	<50 Gy	REF			
					
OS					
Histology	Oligodendroglioma	0.37 (0.22-0.63)	<.001	0.42 (0.21-0.86)	.018
	Astrocytoma	0.53 (0.33-0.87)	.013		NS
	Glioblastoma	REF		REF	
ECOG PS	0	0.58 (0.37-0.90)	.016		NS
	≥1	REF			
Time to recurrence	>60 mo	0.41 (0.25-0.68)	<.001	0.49 (0.28-0.87)	.014
	<60 mo	REF		REF	
PT-ReRT dose	≥50 Gy	0.55 (0.37-0.83)	.004		NS
	<50 Gy	REF			

*Abbreviations:* PFS = progression-free survival; CNS = central nervous system; ECOG PS = Eastern Cooperative Oncology Group performance status; HR = hazard ratio; PT-ReRT = proton therapy reirradiation; WHO = World Health Organization.

**Figure 3:**
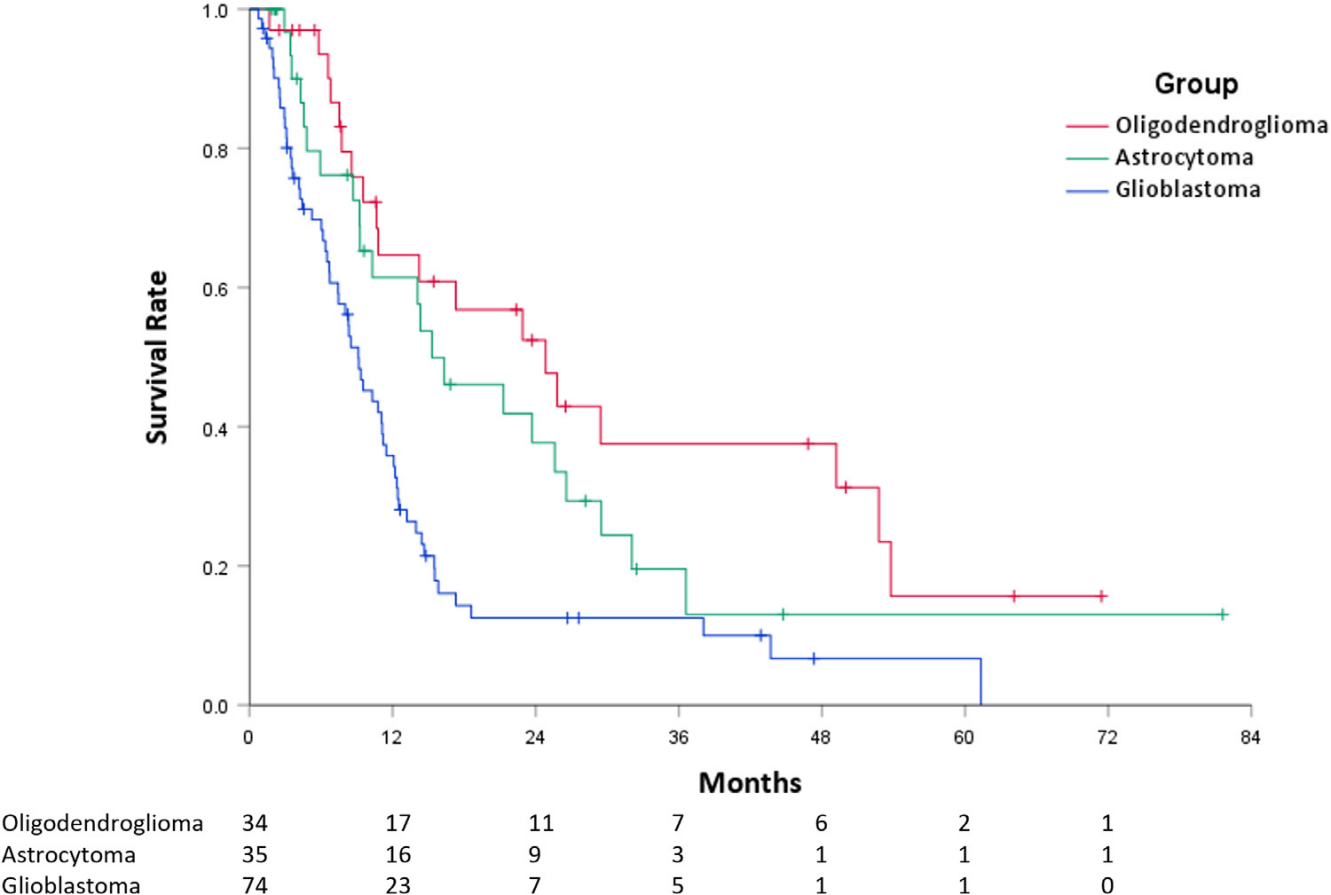
Kaplan-Meier curves demonstrating overall survival by original histology (red = oligodendroglioma, green = astrocytoma, blue = glioblastoma).

The median OS after PT-ReRT was 11.2 months (95% CI, 9.0-13.4). Median OS was longer for patients with oligodendroglioma (24.8 months, 95% CI, 12.9-36.8) and astrocytoma (15.3 months, 95% CI, 6.7-23.9) histologies compared to glioblastoma (9.1 months, 95% CI, 7.0-11.3; *P* < .001) (Fig. 3). The median OS from time of initial diagnosis for the entire cohort, oligodendroglioma, astrocytoma, and glioblastoma histologies was 86.7 months (95% CI, 57.3-116.2), 209.0 months (95% CI, 154.5-263.5), 122.5 months (95% CI, 73.0-172.0), and 47.3 months (95% CI, 33.4-61.2), respectively. On UVA, improved OS after PT-ReRT was associated with oligodendroglioma (HR 0.37, 95% CI, 0.22-0.63, *P* < .001) and astrocytoma (HR 0.53, 95% CI, 0.33-0.87, *P* = .013) histologies compared to glioblastoma, ECOG performance status 0 (HR 0.58, 95% CI, 0.37-0.90, *P* = .016), time interval since prior RT >60 months (HR 0.41, 95% CI, 0.25-0.68, *P* < .001), and ReRT dose ≥50 Gy (HR 0.55, 95% CI, 0.37-0.83, *P* = .004). Surgery within 3 months was not found to be significant for OS (HR 0.78, 95% CI, 0.49-1.23, *P* = .28). On MVA, improved PFS remained significant for oligodendroglioma (HR 0.42, 95% CI, 0.21-0.86, *P* = .018) and time since prior RT >60 months (HR 0.49, 95% CI, 0.28-0.87, *P* = .014) (Table 2).

### Toxicity

At baseline just before PT-ReRT, 12 patients (9%) had documented grade 3 toxicities from prior therapies. Acute grade 2+ and grade 3+ toxicities possibly, probably, or definitely related to PT-ReRT were reported in 45% and 8% of patients, respectively. Acute grade 3 toxicities included ataxia (*n* = 3), fatigue (*n* = 1), headache (*n* = 3), weakness (*n* = 5), neuropathy (*n* = 1), and seizure (*n* = 1). On UVA, the only variable associated with acute grade 3 toxicity was ECOG 2 to 3 (HR 16.15, 95% CI, 3.0-86.8, *P* = .0012); specifically, cumulative dose was not associated with acute toxicity.

Of the 110 patients who had >3 months follow-up, late grade 2+ and grade 3+ toxicities occurred in 17 (15%) and 4 (4%) patients, respectively. Late grade 3 toxicities included muscle weakness (n = 2), memory impairment (n = 1), ataxia (n = 1), dysarthria (n = 1), peripheral neuropathy (n = 1), and seizure (n = 1). No grade 4 to 5 toxicities were observed. Of the 4 patients with late grade 3 toxicities, the median dose of PT-ReRT and cumulative dose of all RT courses were 51.9 Gy and 110.0 Gy, respectively. Individual toxicity figures are detailed in Table 3. Site-defined radiographic radiation necrosis (CTCA grade 1) was reported in 19% of patients out of 100 who had post-ReRT MRI results available for analysis; data regarding symptomatic radiation necrosis (grades 2-5) were not available. The median dose of PT-ReRT and cumulative dose of RT courses for these patients were 57.4 Gy and 114.7 Gy (EQD2, *α*/*β* = 2.5), respectively; on UVA, cumulative dose was not associated with radiation necrosis.

**Table 3:** Acute and late toxicity graded using CTCAE version 4.0

	Acute (*n* = 126)			Late (*n* = 110)		
Toxicity/grade	1	2	3	1	2	3
Alopecia	52 (41)	11 (9)	0 (0)	2 (2)	1 (1)	0 (0)
Anorexia	20 (16)	1 (1)	0 (0)	2 (2)	2 (2)	0 (0)
Ataxia	10 (8)	10 (8)	3 (2)	3 (3)	3 (3)	1 (1)
Blurred vision	20 (16)	2 (2)	0 (0)	1 (1)	2 (2)	0 (0)
Confusion	21 (17)	4 (3)	0 (0)	2 (2)	3 (3)	0 (0)
Dermatitis	59 (47)	2 (2)	0 (0)	1 (1)	0 (0)	0 (0)
Dizziness	17 (13)	5 (4)	0 (0)	0 (0)	0 (0)	0 (0)
Dysarthria	10 (8)	2 (2)	0 (0)	2 (2)	2 (2)	1 (1)
Fatigue	44 (35)	19 (15)	1 (1)	6 (5)	5 (5)	0 (0)
Headache	19 (15)	5 (4)	3 (2)	3 (3)	3 (3)	0 (0)
Insomnia	13 (10)	8 (6)	0 (0)	0 (0)	2 (2)	0 (0)
Memory impairment	3 (2)	3 (2)	0 (0)	0 (0)	2 (2)	1 (1)
Muscle weakness	7 (6)	12 (10)	5 (4)	2 (2)	7 (6)	2 (2)
Nausea	19 (15)	3 (2)	0 (0)	3 (3)	0 (0)	0 (0)
Peripheral neuropathy	8 (6)	3 (2)	1 (1)	1 (1)	1 (1)	1 (1)
Seizure	0 (0)	2 (2)	1 (1)	4 (4)	5 (5)	1 (1)
Vomiting	7 (6)	1 (1)	0 (0)	1 (1)	0 (0)	0 (0)
Weight loss	3 (2)	1 (1)	0 (0)	0 (0)	0 (0)	0 (0)
Overall	120 (95)	57 (45)	10 (8)	25 (23)	17 (15)	4 (4)
Radiation necrosis						
Radiographic	19% (19/100)					
Symptomatic	Data not available					

*Abbreviations:* CTCAE = National Cancer Institute Common Terminology Criteria for Adverse Events.

## Discussion

In the current study, we report outcomes of recurrent malignant glioma treated with PT-ReRT. To our knowledge, this multi-institutional, prospectively collected study represents the largest cohort of glioma patients treated with PT-ReRT to date. Although recurrent glioma is a challenging clinical scenario where most patients are heavily pretreated with chemotherapy and high-dose RT, commonly in the range of 54 to 60 Gy, the clinical outcomes of this cohort compare favorably to contemporary photon ReRT series, particularly for nonglioblastoma tumors, and the toxicity profile notably remains acceptable. Additionally, this study corroborates several known prognostic factors of recurrent malignant glioma, including tumor histology, performance status, and time to recurrence.

Several retrospective series of photon ReRT for recurrent glioma have been published, particularly for recurrent CNS WHO grade 4 glioblastoma.^12^^,^^13^ Most series utilize modest dose regimens ranging from 30.0 to 7.5 Gy/10 to 15 fractions (32.5-39 Gy EQD2) and demonstrate relatively consistent rates of guarded survival with low rates of high-grade toxicity.^14^, ^15^, ^16^, ^17^, ^18^, ^19^, ^20^ Combs et al^14^ included 172 patients with malignant glioma treated with a median dose of 36 Gy/13 fractions and reported median OS of 8 months, 16 months, and 22 months for WHO grade 4, 3, and 2 tumors, respectively. OS was associated with both histology and time to recurrence, consistent with our analysis. Fogh et al^15^ reported 147 patients with recurrent glioma undergoing reirradiation to 35 Gy/10 fractions. Median age for this cohort was 53, median OS was 11 months for all patients and 8 months for patients with glioblastoma, and only 1 patient (0.6%) developed late grade 3 toxicity. Although our cohort included favorable prognostic factors such as relatively younger patients and lower grade tumors, possibly biasing outcomes, the aforementioned study reports a very similar patient population and outcomes. The potential benefit of a higher radiation dose was addressed in a contemporary retrospective series reported by Fleischmann et al,^21^ reporting 223 patients with glioma treated with ReRT. Median age was 49; median OS was 10 versus 8 months for patients treated with >36 Gy versus those treated with a lower dose, which was significantly different on UVA but not on MVA, similar to our analysis, thus leaving the case open for ReRT dose escalation.^21^

Recently, the first prospective, multi-institutional randomized trial studying the role of ReRT for recurrent glioblastoma was published. NRG Oncology/RTOG 1205 was a phase 2 randomized trial comparing bevacizumab alone or bevacizumab with hypofractionated reirradiation to 35 Gy/10 fractions (39.4 Gy EQD2). Although the trial did allow PT-ReRT, only 2 patients treated with protons were enrolled. The reirradiation cohort demonstrated a clear PFS benefit (7.1 months vs 3.8 months) but no advantage in OS (10.1 months vs 9.7 months). Toxicity after ReRT was notably low, with acute and late grade 3+ events for the whole cohort of 5% and 0%, respectively.^22^ Another randomized trial was recently conducted by Henry Ford Hospital comparing bevacizumab alone to bevacizumab with fractionated radiosurgery to 32 Gy/4 fractions (48 Gy EQD2) with similar clinical and toxicity outcomes.^23^ The current cohort compares favorably with these contemporary results; median OS and PFS were 11.2 and 8.1 months, respectively, with a relatively high median ReRT dose of 44.6 Gy EQD2.

Reports of PT-ReRT for recurrent malignant glioma are limited and considerably smaller than the current analysis. Mizumoto et al^20^ described 26 patients with recurrent malignant brain tumors, including 8 who received PT. The median ReRT dose and OS of patients with glioblastoma (n = 5) were 30 Gy EQD2 and 18.3 months, respectively. No high-grade toxicities were reported.^20^ Galle et al^24^ reported on 20 patients with recurrent malignant glioma retreated with PT. Median dose and OS for patients with grade 3 and 4 tumors were 59.4 Gy EQD2/24.9 months and 54 Gy EQD2/7.8 months, respectively. Two patients developed radiation necrosis, and only one of them was symptomatic.^24^ Lastly, Saeed et al^25^ reported previously on a subset of PCG patients using PT to treat recurrent glioblastoma, including 45 patients receiving a median dose of 46.2 Gy EQD2 (range, 25-60 Gy EQD2). The median PFS and OS were encouraging at 13.9 and 14.2 months, respectively.^25^ While results for our glioblastoma patients appear consistent with these historical series, the outcomes of patients without glioblastoma from the current series compare favorably with median OS for grade 2/3 astrocytoma and oligodendroglioma of 23.7 months and 43.7 months, respectively. Coupled with the low OARs dose achieved with protons, PT-ReRT may present an attractive treatment option for this favorable patient population.

Regarding toxicity, the overall incidence of acute grade 3 toxicity in our study was 7%, while late grade 3 toxicity was 4%. There were notably no grade 4 to 5 PT-related events. These rates are generally higher than the previously described trials including Combs et al^14^ that had no grade 3+ toxicities and NRG Oncology/RTOG 1205 that reported acute and late toxicity rates of 5% and 0%, respectively.^22^ These rates are may be attributable to the higher median dose used in this cohort relative to the existing photon reirradiation literature, consistent with the study by Saeed et al^25^, which reported a late grade 3 toxicity of 8.3% after a median reirradiation dose of 46.2 Gy EQD2. However, higher PT-ReRT dose was not associated with grade 3 toxicity in this analysis; the only associated variable was ECOG 2 to 3, providing an alternative explanation for the higher toxicity rates. In addition, the overall incidence of radiographic radiation necrosis was 19%, similar to the 21% recently reported by Fleischmann et al^21^, with no association to higher ReRT doses. Unfortunately, rates of symptomatic radiation necrosis were not recorded separately for this cohort; nonetheless, it is reassuring that the overall rate of significant toxicity appears to remain modest.

Based on these outcomes, lower dose hypofractionated reirradiation is the most appropriate regimen for most patients, particularly those with high-grade recurrences in whom quality of life is prioritized and tolerance for treatment-related toxicity is low. Higher reirradiation doses may be contemplated mainly for patients with more favorable prognoses, as they have been associated with improved clinical outcomes both in this study (albeit, only on UVA) and prior series.^17^^,^^26^, ^27^, ^28^, ^29^, ^30^ Further investigation of dose escalation in the recurrent setting should be conducted thoughtfully on a clinical trial in well-selected patients, such as those with a lower grade recurrence, good performance status, and a prolonged disease-free interval following the initial course of RT.

Despite the use of a prospectively collected multi-institutional data set, several limitations to the current work are notable. Lack of molecular data, particularly for patients without glioblastoma, precluded a more accurate stratification of the analysis; WHO classification itself was also modified during the study duration, incorporating IDH status which was not available for the majority of our patients. In addition, the database included patients treated for both first and multiple recurrences, was unable to collect patterns of recurrence, and lacked detailed dosimetric parameters, including extent of field overlap and OARs doses. These specific factors are critical to optimal patient selection and more detailed outcomes analyses. More robust prospective evaluations of these factors will be needed on future prospective institutional trials. Lastly, PCG toxicity data rely on local institutional evaluation, and the database does not provide primary radiographic data. Therefore, rates of radiographic radiation necrosis and estimates of PFS were based on reports from the individual institutions and were not subject to a central review to distinguish adverse treatment effects from disease progression. Moreover, rates of symptomatic radiation necrosis were not recorded, significantly limiting any conclusion regarding the incidence of higher-grade adverse events in this patient population and warranting close toxicity monitoring in any future study of proton reRT. Some patients did not continue regular follow-up at their proton center of treatment, potentially underestimating certain events, especially late toxicities. Finally, patient selection was made at the discretion of each individual center without prespecified criteria, which may result in selection bias.

In conclusion, this series consists of the largest multi-institutional prospectively collected cohort of patients with recurrent malignant glioma treated with PT-ReRT to date. Efficacy outcomes appeared favorable, particularly for patients without glioblastoma, with modestly higher rates of toxicity relative to photon series in the context of escalated median PT-ReRT dose. Further studies are warranted to prospectively investigate the potential advantages and risks associated with PT-ReRT and quantify potential neurocognitive and quality-of-life differences.

## Disclosures

None.
